# A Simple Approach for Regenerating Electrolyzed Hydrogen Production Using Non-De-Ionized Water Sources

**DOI:** 10.3390/ma16237382

**Published:** 2023-11-27

**Authors:** Wei-Hsiang Chiang, Shiow-Jyu Lin, Jong-Shinn Wu

**Affiliations:** 1 College of Photonics, National Yang Ming Chiao Tung University, Tainan 71150, Taiwan; x010203042003@gmail.com; 2 Department of Electronic Engineering, National Ilan University, Ilan 260007, Taiwan; 3 Department of Mechanical Engineering, National Yang Ming Chiao Tung University, Hsinchu 30010, Taiwan

**Keywords:** hydrogen energy, proton exchange membrane fuel cell, hydrogen production, filtration system

## Abstract

This research focuses on using natural renewable water resources, filters, and performance recovery systems to reduce the cost of generating pure hydrogen for Proton Exchange Membrane Fuel Cells (PEMFCs). This study uses de-ionized (DI) water, tap water, and river water from upstream as the water source. Water from these sources passes through 1 μm PP filters, activated carbon, and reverse osmosis for filtering. The filtered water then undergoes hydrogen production experiments for a duration of 6000 min. Performance recovery experiments follow directly after hydrogen production experiments. The hydrogen production experiments show the following: DI water yielded a hydrogen production rate of 27.13 mL/min; unfiltered tap water produced 15.41 mL/min; unfiltered upstream river water resulted in 10.03 mL/min; filtered tap water yielded 19.24 mL/min; and filtered upstream river water generated 18.54 mL/min. Performance recovery experiments conducted by passing DI water into PEMFCs for 15 min show that the hydrogen generation rate of tap water increased to 25.73 mL/min, and the rate of hydrogen generation of upstream river water increased to 22.58 mL/min. In terms of cost-effectiveness, under the same volume of hydrogen production (approximately 600 kg/year), using only DI water costs 1.8-times more than the cost of using filtered tap water in experiments.

## 1. Introduction

Hydrogen is a clean source of energy [1,2]. Using hydrogen as fuel only results in the production of water. Only producing water reduces the emission of greenhouse gases and also decreases air pollution. Hydrogen stores a large amount of energy. Most energy production methods face the issue caused by peak and off-peak hours. Storing excess energy production during off-peak hours in the form of hydrogen increases energy usage efficiency.

Three of the most notable methods of industrial hydrogen production include hydrogen production from fossil fuels [3,4], electrolysis [5,6], and PEMFC reversal [6,7,8,9]. Hydrogen production from fossil fuels requires non-renewable resources, and electrolysis possesses low conversion efficiency. Both of the above methods require the use of purifiers to increase the purity of hydrogen. Even then, the purified gasses still have a hydrogen purity of less than 5N (99.999%). The purity of hydrogen gas significantly affects the longevity and efficiency of power generators. Meanwhile, PEMFC reversal can not only produce hydrogen above level 5N but it also produces only water as a byproduct. In addition, PEMFC reversal has a high conversion efficiency, produces no harmful chemicals or pollution, and requires very little space. This process shows an enormous amount of potential for future hydrogen production.

The three major components of a PEMFC [10] include electrodes, electrolyte membrane, and bipolar plates. The core component of a PEMFC is a membrane electrode assembly (MEA), composed of a Proton Exchange Membrane (PEM) sandwiched between the cathode and anode. PEM [11,12] is an ionized polymer membrane capable of conducting protons with a porous solid film structure, providing atomic ion channels. The channels’ primary functions include: (1) delivering H^+^ or OH^-^ ions, and (2) separating hydrogen and oxygen to reduce safety hazards. The manufacturing of electrodes [13] requires spreading a layer of a catalyst made from carbon powder and platinum on a sheet of carbon fiber fabric or carbon fiber paper. The outer layer is a diffusion layer [14]. This layer allows the reacting gases to pass through uniformly. The diffusion layer also allows water to be directed outward during the forward reaction and uniformly directed inward during the reverse reaction. This layer provides structural support and also increases the reaction area of catalysts. The inner layer is the catalyst layer [15], which is where electrochemical reactions occur.

Generally, catalysts utilize carbon black as the carrier to increase the reaction surface area and reduce platinum usage to decrease the cost. In addition, bipolar plates [16] are located outside of the MEAs. These plates provide adequate gas sealing to separate oxidants and reductants. The sealing prevents gases from diffusing, causing a short circuit. During forward reactions, the sealing has the effect of collecting current. The channel design within the plates also affects the uniformity of reactant distribution to the electrodes. The contemporary method for generating hydrogen for PEMFCs is through the electrolysis of DI water. Nevertheless, the cost and complexity of manufacturing DI water make its widespread use challenging. In addition, using other readily accessible water sources, e.g., tap water and even river water in some rural areas, may popularize the use of PEMFCs for producing hydrogen. However, it could clog up the membrane in PEMFCs, decreasing the hydrogen production rate or even damaging the device. Thus, how to design a simple process by regenerating the MEAs efficiently while using readily accessible water sources may become an effective approach to popularize the use of PEMCFs as the source of hydrogen.

Therefore, this research developed a system capable of producing highly purified hydrogen for PEMFCs using readily available water sources. Furthermore, after analyzing water samples filtered with simple filtering equipment using inductively coupled plasma mass spectrometry (ICP-MS), the results show that inorganic matter in the water significantly affected the experiments. Using filtered water and cleaning the membrane at specific times effectively decrease the rate of PEMFC clogging. The organization of this paper includes the Research Method, then the Results and Discussion, and, finally, the major considerations in the Section 4.

## 2. Research Methods

### 2.1. Overview

This research studies the effects of tap water and upstream river water on hydrogen production rates with DI water as the control. DI water continuously produces hydrogen throughout the entire experiment without special processing. Both filtered and unfiltered tap water and river water undergo hydrogen production experiments for a hundred hours to study the decay of the hydrogen production rate over time. Furthermore, filtered water sources undergo three cycles of hydrogen production and performance recovery experiments to study the change in hydrogen production efficiency. Comparing the results of the above studies yields an excellent benchmark of the cost-effectiveness of using water sources other than DI water.

### 2.2. Water Filtering and Filtration Equipment

**Figure 1:**
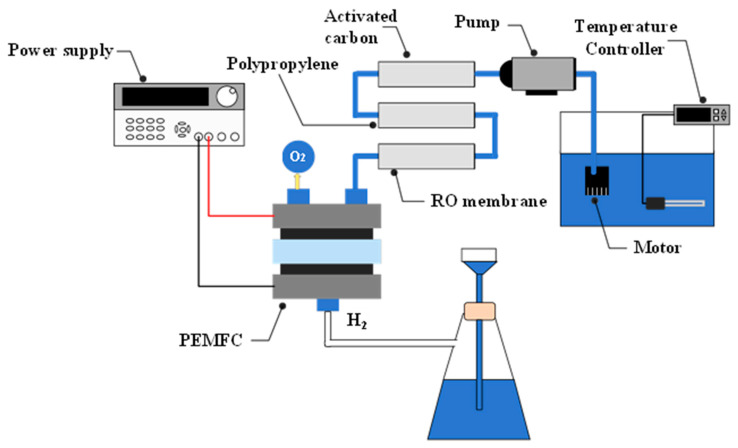
Water filtering device setup.

Figure 1 shows the process of generating hydrogen from filtered water sources. The first-stage filter includes activated carbon that uses the micropores to achieve adsorption. Activated carbon is commonly manufactured from green oak, bamboo, coconut shells, or Japanese cedar; the primary function of activated carbon in this experiment is to remove the chlorine in tap water as chlorine erodes the membrane used for reverse osmosis (RO). The second-stage filter is made from meltblown polypropylene fiber filter solids of larger diameters, such as sand, rust, and gelatinous matter. The pores in this filter are one to five micrometers in diameter. The third-stage filter solids of are composed of smaller diameters using reverse osmosis. Reverse osmosis effectively decreases the total dissolved solids (TDS), including bacteria and various salts. As a result, RO can achieve a rate of desalination up to 95%. After filtering, water passes through PEMFCs to test their hydrogen production capabilities. This study then compares the results of using filtered versus unfiltered tap and river waters for hydrogen production.

### 2.3. Hydrogen Production Experiment

**Figure 2:**
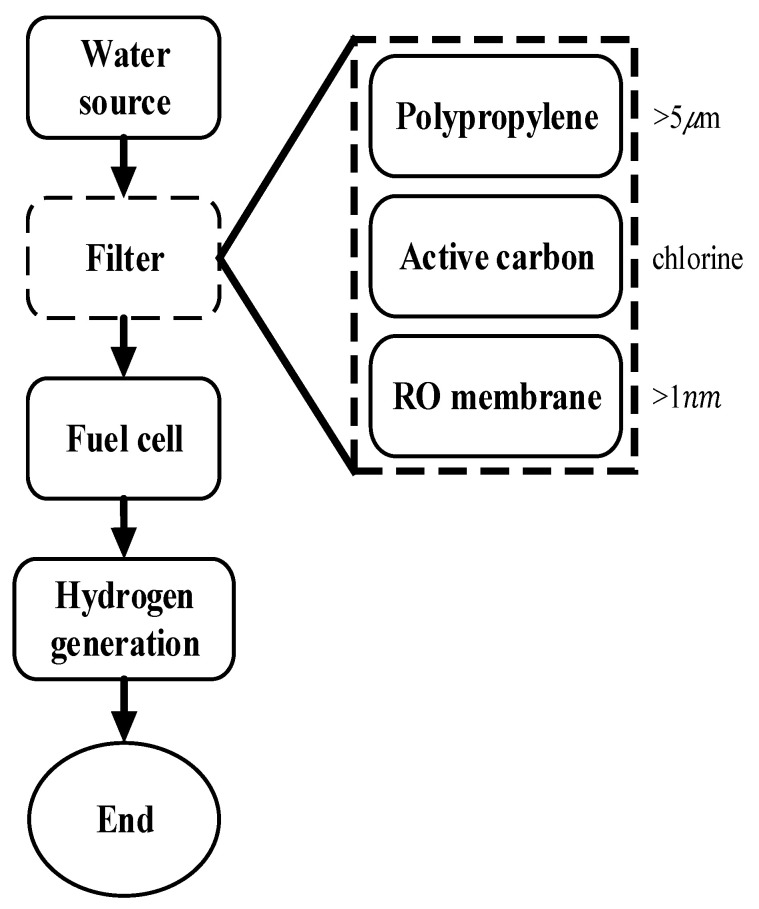
Flowchart of hydrogen production experiment.

To conduct this section of the experiment, first, activate PEMFCs with DI water for fifteen minutes. Then, only pass through the experimental water source after the rate of hydrogen production has stabilized. Figure 2 shows the process of the hydrogen production experiment. The experiment uses a 2.5 V 4 A direct current for one hundred hours. The upstream water maintains a temperature of 25 degrees Celsius by using a thermostat. The experiments follows this order: DI water, unfiltered tap water, then unfiltered upstream river water. Experiments studying the effects of filtered water follow the same order.

### 2.4. Performance Recovery Experiment

**Figure 3:**
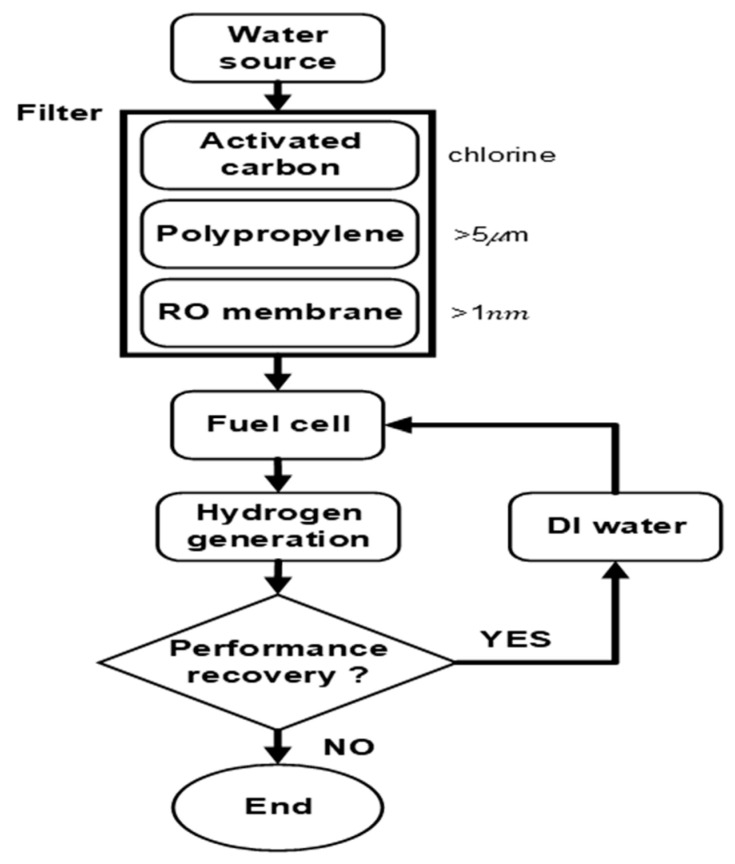
Flowchart of hydrogen production using filtered water sources.

Figure 3 shows the experimental process of the performance recovery experiment. First, the system undergoes a cleansing process using DI water. After injecting DI water into the experimental setup and sitting for thirty minutes for cleaning, the system then undergoes ten hours of performance recovery testing. The rate of damage that PEMFCs experience can be estimated by first conducting performance recovery and then regenerating hydrogen generation using the same PEMFCs. The rate of hydrogen production generally recovers after conducting the performance recovery stage. Therefore, the hydrogen production experiment can be performed repeatedly. The same logic applies to other performance recovery experiments. This study repeated this cycle of hydrogen production and performance recovery experiments three times for comparison purposes.

### 2.5. Water Sample Analysis Using ICP-MS

This research uses ICP-MS to analyze the chemical composition of various water sources. The experiments utilize the Thermo Fisher Scientific (Waltham, MA, USA) iCAP TQ to conduct ICP-MS. To prepare the samples for testing, prepare a reagent using 15 mL of super-pure nitric acid and DI water. Then, prepare a standard solution using standard reference materials of the tested element. After completing the above steps, form the test solution using the standard solution and 15 g of the test sample. Also, prepare a blank solution using the standard solution and DI water. Lastly, move the solutions above into storage bottles for testing. The test determines the concentration of each element using the calibration curves generated during the experiment.

## 3. Results and Discussion

### 3.1. Hydrogen Production Experiment

**Figure 4:**
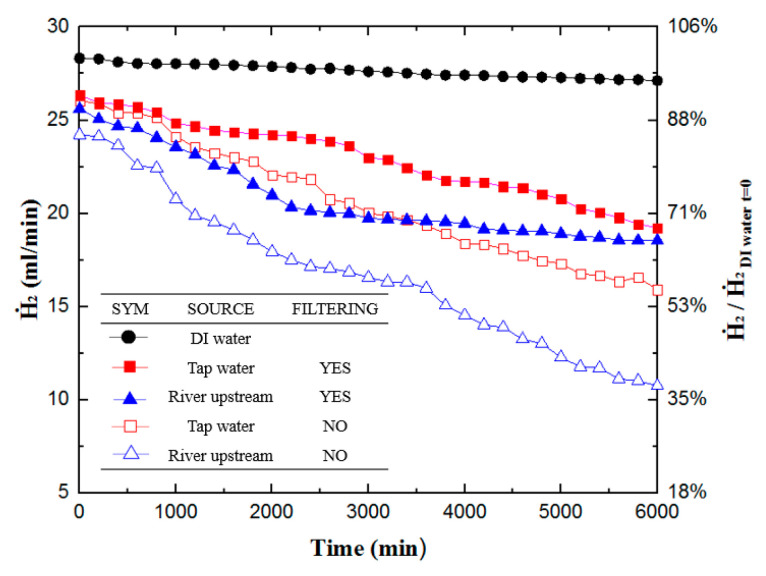
Hydrogen production rates of various water sources hydrogen production experiment with different water sources. The *x*-axis is the time from 0 to 6000 min. The left vertical axis is the hydrogen production rate (mL/min), and the right vertical axis is the hydrogen production rate relative to the hydrogen production rate of DI water at 0 s.

Figure 4 shows the results of hydrogen production experiments for DI water, unfiltered/filtered tap waters, and unfiltered/filtered river upstream waters. During the 6000 min hydrogen production experiment, DI water had the best steady production curve, the highest hydrogen production rate, reaching 28.33 mL/min at the beginning, and 27.13 mL/min at the end of 100 h. Therefore, this study uses the results of this test as the baseline value (100%) for comparing with other hydrogen production experiments.

Experiments always yield the highest rate of hydrogen production in the initial stage. In terms of initial hydrogen production rate, filtered tap water shows the most potential, with a rate of 93%, while unfiltered tap water reaches 91%, filtered upstream river water reaches 90.4%, and unfiltered upstream river water reaches 87.6%. In comparison to the other samples, filtered tap water has the minutest impurities. Thus, its initial hydrogen production rate approaches that of DI water. On the other hand, the worst performing sample is the unfiltered upstream river water. This result indicates that the upstream river water has the highest impurity content among all the samples, including organics, heavy metals, bacteria, etc., leading to lower hydrogen production rates, even at the beginning of the experiment. Figure 4 shows that filtered river water has a lower hydrogen production rate than unfiltered tap water in the first 3000 min. However, its production rate plateaus after 2000 min and surpasses unfiltered tap water at the 3000 min mark for the rest of the experiment. Each sample’s terminal hydrogen production rate at the end of the experiment is as follows: DI water yields a hydrogen production rate of 95.8% (27.13 mL/min); filtered tap water yields a rate of 68% (19.24 mL/min); unfiltered tap water ended up with 54.4% (15.41 mL/min); filtered upstream river water yields a rate of 65.4% (18.54 mL/min); and unfiltered upstream river water has a rate of 35.4% (10.03 mL/min). In fact, the novelty of this approach may lie in the fact that the performance of unfiltered water is not too bad, and considering the cost, it could be a viable alternative. However, it is essential to note that over an extended period, the accumulation of contaminants may necessitate the replacement of components.

### 3.2. Performance Recovery Experiment

**Figure 5:**
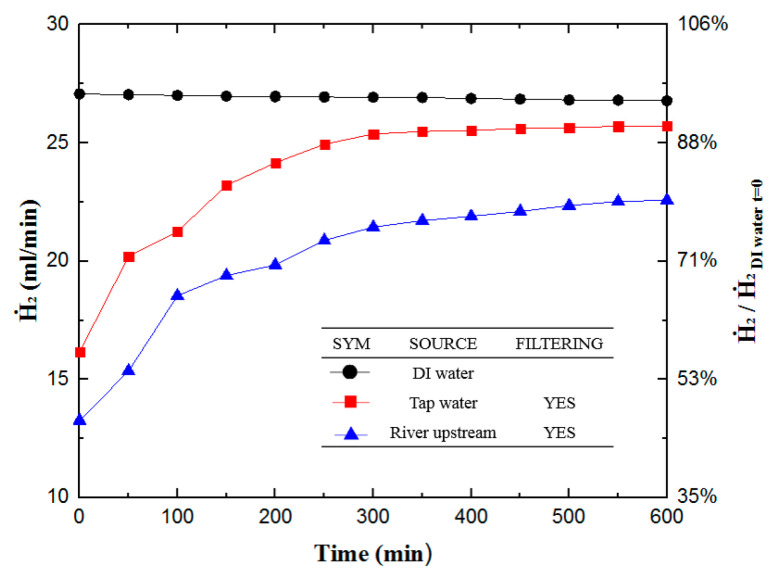
Results of performance recovery experiments for filtered water sources except DI water. Hydrogen production experiments with different water sources (DI water for reference).

Figure 5 shows the efficiency of performance recovery of PEMFCs for 600 min after hydrogen production for water sources, including DI water, filtered upstream river water, and filtered tap water. Each data point was obtained by averaging three sets of experiments. After 6000 min of hydrogen production experimentation, all the samples undergo performance recovery testing. The first step of the experiment is to extract all water from the PEMFCs. Then, DI water passes through the used PEMFCs for 15 min to activate the PEMFCs. Once the hydrogen production rate reaches an equilibrium, the PEMFC then undergoes performance recovery experimentation for 600 min. In practice, the membrane was nearly recovered after 300 min, which the production rate saturated afterwards.

Cleansing the fuel cell using DI water does not fully recover the performance of the PEMFC, as the impurities within the water have already contaminated the membrane. Nevertheless, passing DI water through the used PEMFC does recover some of the performance losses. The hydrogen production rate throughout first increases then plateaus at the 300 min mark. After the experiments, the hydrogen production rate of each recovered sample is as follows: the filtered tap water sample reaches a rate of 25.73 mL/min, 90.8% of the reference value; the filtered upstream river water sample reaches a rate of 22.58 mL/min, approximately 79.7% of the reference value. Overall, the filtered tap water sample recovered 22.8% of its generation capacity, while the filtered upstream river water recovered 14.3%.

### 3.3. Results after Three Cycles of Hydrogen Production and Performance

#### Recovery Experiment

**Figure 6:**
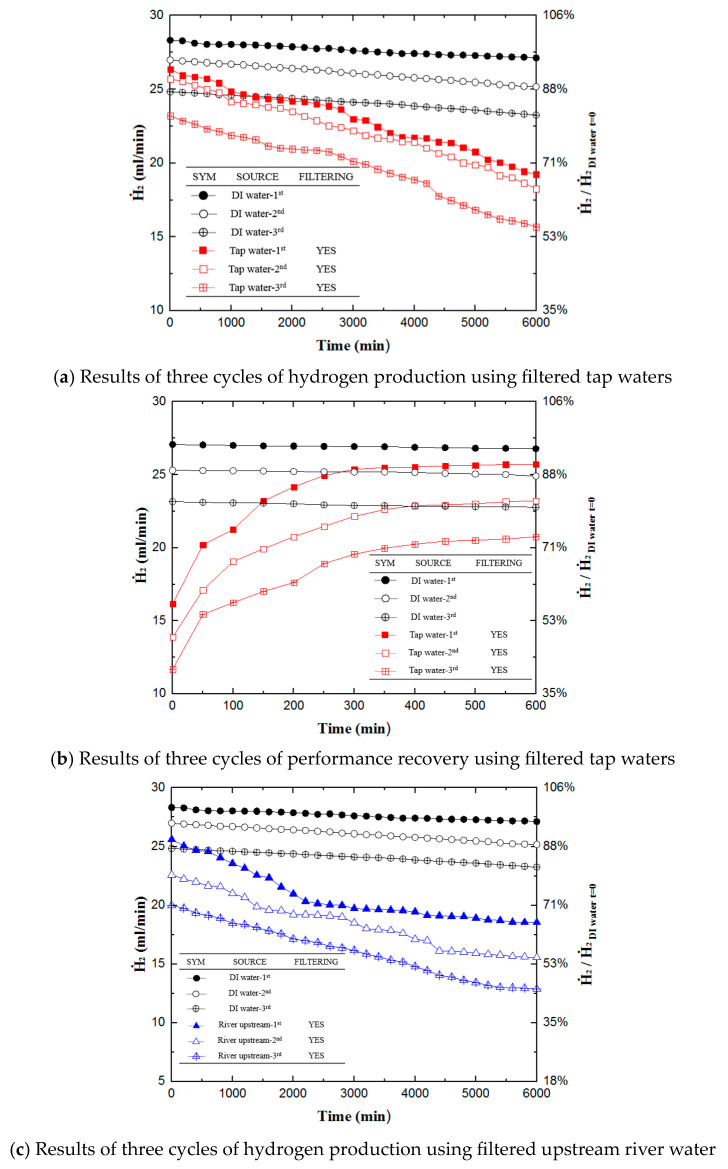

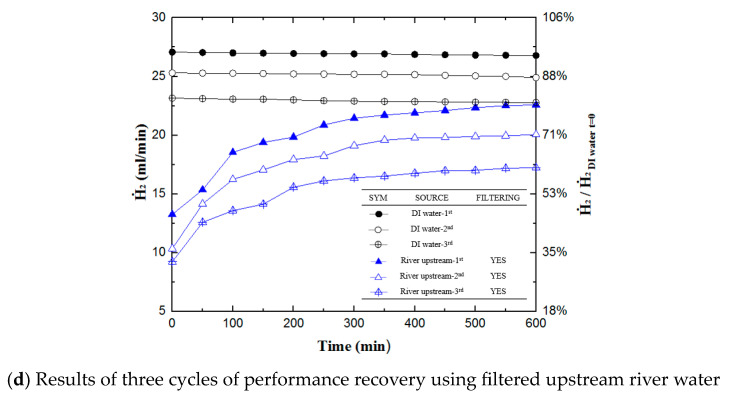
Summary of the three cycles of experiments conducted using various samples. (**a**) Hydrogen generation of filtered tap water over three cycles of experimentation; (**b**) performance recovery of filtered tap water over three cycles of experimentation; (**c**) hydrogen generation of filtered upstream river water over three cycles of experimentation; (**d**) performance recovery of filtered upstream river water over three cycles of experimentation.

After carrying out performance recovery, hydrogen production takes place again. The combination of one generation and one recovery experiment constitutes “one cycle” of an experiment. Three cycles of experiments were performed throughout this research with each sample to study the effect of repeated hydrogen generation and performance recovery. As shown in Figure 6, after three cycles of experiments totaling 19,800 min, DI water resulted in a hydrogen production rate of 22.78 mL/min, 80% of the reference value. Filtered tap water resulted in 20.78 mL/min, 73.2% of the reference value. Filtered upstream river water yielded 17.25 mL/min, 60.9% of the reference value. Nevertheless, the production decaying rate was higher for the non-DI water source. This research demonstrates that the method used for performance recovery has the capability of being repeated efficiently. Both chemical performance decay caused by carbon monoxide and physical performance decay caused by impurities clogging up the membrane resulted in the overall decrease in the performance of PEMFCs. This research recovers the physical performance of the fuel cells by washing out the impurities deposited in the membrane by simply feeding the DI water through the membrane for 600 min, which is very easy in practical operation.

### 3.4. Water Sample Analysis Using ICP-MS

**Figure 7:**
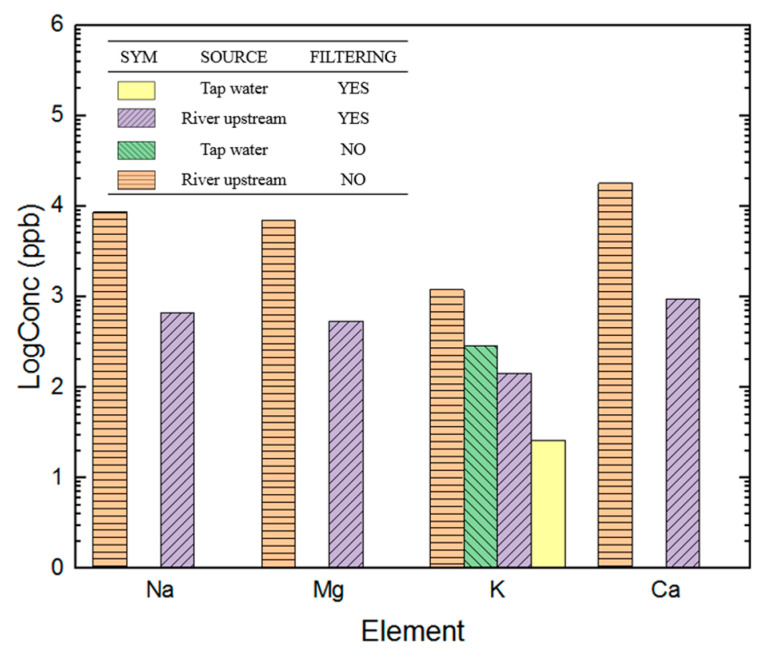
Measured concentrations of elements in different water samples.

As shown in Figure 7, the amount of inorganic matter in the water decreased significantly after filtering, especially Na, Ca, K, and Mg for the river upstream water. All of the inorganic materials decreased by 90% or one order of magnitude. Upstream river water contains a large amount of sodium, mainly from the dissolution of minerals. Sodium only possesses one electron in its outer orbit, meaning that it loses the electron quickly, forming Na^+^. Having Na^+^ in water does not suit the requirement for hydrogen production using electrolysis. Both K and Mg are metal, with K carrying one positive charge. Due to having similar ionizing energies, the atom gives away its only electron in the outer orbit. The characteristics of the inorganic matter present in water sources show that they all cause hydrogen production to decrease in PEMFCs, regardless of their electrical charge. Hydrogen generation using PEMFCs benefits from the fact that pure water is not electrolytic. After reactions, crystals formed by inorganic matter may deposit on the surface of the diffusion layer of the catalyst layer, stopping the diffusion of gases and reducing the surface area available for electrolysis, which definitely requires further investigation.

### 3.5. Cost-Effectiveness Analysis

DI water has an expiration date after its production. If not used immediately or stored under non-immaculate conditions, impurities from the air or the surroundings may dissolve into DI water. The requirement of using DI water is why popularizing the use of PEMFCs becomes difficult in practice. The method proposed by this paper only requires a small amount of DI water for performance recovery to achieve the desired results. Table 1 analyzes the cost-effectiveness of both options using DI water and filtered tap water as an example. Maintenance and repair cost approximately one-tenth of the cost of building. The equipment used for DI water production is GenPure XCAD UV-TOC, with a Nafion 211 PEM. This research studied the cost-effectiveness analyses of running the system for one year and five years.

Although the hydrogen production rate using methods proposed in this research cannot match that of DI water, the methods proposed in this research demonstrate high economic potential, and it might become more suitable for promoting the development of the hydrogen energy industry. As shown in Table 1, under the assumed hydrogen generation rate of 600 kg/year, the DI water approach costs more than this proposed method. The proposed method requires more PEMFCs than DI water, causing the cost in the first year to approach that of DI water. However, most of the decay in a PEMFC is a result of carbon monoxide contamination, as pointed out in [17,18]. Therefore, replacing PEMs restores the PEMFC’s efficiency. According to other studies [19,20], replacing the PEMs when the hydrogen production rate is down to 40% yields the optimal result. The cost-effectiveness study shows that the cost of hydrogen production using DI water is 1.8-times larger than the method proposed in this research by the fifth year.

## 4. Conclusions

After a long duration of hydrogen production experimentation, the results show that the impurities in water sources, especially inorganic matter, and salts significantly affect PEMFCs when non-DI water sources are used. The results also demonstrate that DI water has the highest hydrogen production efficiency, as expected. However, the cost of the equipment and maintenance needed for DI water production is high, making using hydrogen as an acceptable energy source challenging. Therefore, this research introduces a simple method that first uses common filtered water sources to generate hydrogen, then only uses DI water to recover loss performance. The process can be repeated for many cycles depending upon the user’s decision, while it is demonstrated three times in this study. The major findings of this research are summarized as follows:

(1) The experiment utilizing low-cost water sources, such as upstream river water and tap water, to generate hydrogen using PEMFCs shows that filtered water sources have a higher hydrogen generation output than unfiltered water sources. From the hydrogen output data towards the end of the experiment, the hydrogen production of filtered tap water increased by 13.6% and that of filtered upstream river water improved by 30%.
(2) After the first 6000 min hydrogen production experiment, performance recovery for 600 min using water showed that the filtered tap water sample recovered 22.8% of its efficiency, while the filtered upstream river water recovered 14.3%.
(3) The method proposed in this research allows for repeated usage of the fuel cell, and even non-DI water sources are used. This research conducted three cycles of hydrogen generation and performance recovery experiments. After the final cycle of experimentation, filtered tap water yielded a hydrogen generation rate of 73.2%, while the filtered upstream river water produced a rate of 60.9%. Note that the hydrogen production rate of DI water decreased to 80%.
(4) The method proposed by this research shows significant potential in promoting the usage of PEMFCs. Compared to the usage of DI water, using filtered water and performance recovery significantly reduces the cost of production. Under the exact production rate requirement (600 kg/year), the total cost of producing hydrogen with DI water after five years is 1.8-times that of the method proposed in this research.

## Figures and Tables

**Table 1:** Cost-effectiveness analysis (hydrogen generation rate 600 kg/year) * (unit: USD).

	Method	DI Water (5 Year)	DI Water (1 Year)	Tap Water (5 Year)	Tap Water (1 Year)
Cost					
**NRE**					
Construction *		7765	1553	67	13
Fuel Cell **		2400	2400	4140	4140
**RE**					
Maintenance ***		1017	395	421	415
**Utility bill ^#^**		760	152	179	36
**PEM ^##^**		1553	307	2645	529
**Sum**		13,495	4807	7452	5133

The following is a detailed description of Table 1. **Construction** *: The equipment cost of manufacturing DI water over a five-year duration includes the price of a GenPure XCAD UV-TOC water purifier (USD 7765), a Rio+800 submerged pump for pumping tap water (USD 17), an MOT-8000 pump (USD 33), and other miscellaneous filtering equipment such as activated carbon, water filter, and RO-membranes (USD 17 total). Averaging the total cost over five years yields the cost per year of DI water production. **Fuel Cell** **: DI water requires 40 fuel cells to achieve the same hydrogen production rate (600 kg/year), while tap water requires 69 fuel cells. This study uses the GESL020A fuel cell (USD 60 per fuel cell). **Maintenance** ***: Traditional DI Water incurs a total cost of USD 1017 over five years, averaging USD 203.4 per year. In contrast, this study utilizing Tap Water requires USD 421 over the same period, averaging USD 84.2 annually. This translates to an average yearly savings of approximately 58.6% in consumables costs. **Utility Bill ^#^**: The system requires 6000 L of water per year to generate enough DI water. This amount costs approximately USD 36 per year, based on Taiwan’s cost of water. In addition, the water filtration equipment requires 0.11 kW of power. Assuming 1kWh of electricity costs USD 0.12, the total electricity bill per year costs USD 112. The cost of tap water is negligible due to its low consumption. The pumps require a total of 0.035 kW of power, yielding a yearly cost of USD 36. **PEM** **^##^**: This research uses the Nafion 211 2.5 cm × 2.5 cm membrane (USD 2.6/PEM). DI water requires 120 PEMs per year, while tap water requires 207 PEMs. All of the above calculations were cycled.

## Data Availability

Data are contained within the article.
